# A delayed diagnosis of congenital midline cervical cleft

**DOI:** 10.1002/ccr3.5540

**Published:** 2022-03-10

**Authors:** Adedayo Idris Salawu, Shuaib Kayode Aremu, Babalola Fatai Olakunle, Toye Gabriel Olajide, Abiodun Idowu Okunlola, Ogidi Adetolu Samuel, Kayode Rasaq Adewoye, Chijioke Cosmas Achebe

**Affiliations:** ^1^ Surgery Department College of Medicine and Health Sciences Afe Babalola University Ado‐Ekiti Ekiti State Nigeria; ^2^ ENT Department College of Medicine and Health Sciences Afe Babalola University Ado‐Ekiti Ekiti State Nigeria; ^3^ Sugery Department College of Medicine and Health Sciences Afe Babalola University Ado‐Ekiti Ekiti State Nigeria; ^4^ Community Medicine Department College of Medicine and Health Sciences Afe Babalola University Ado‐Ekiti , Ekiti State Nigeria; ^5^ Radiology Department College of Medicine and Health Sciences Afe Babalola University Ado‐Ekiti Ekiti State Nigeria

**Keywords:** cervical clefts, congenital, midline, sinus, surgery

## Abstract

Congenital midline cervical cleft (CMCC) is an extremely rare anomaly of the neck that typically presents in the neonatal period as a thin suprasternal vertical band of erythematous skin with a nipple‐like projection. We present the management of this uncommon and rarely described entity in a 9‐year‐old girl.

## 1 INTRODUCTION

Congenital midline cervical cleft (CMCC) is an exceedingly rare neck abnormality that often manifests as a thin suprasternal vertical strip of erythematous skin with a nipple‐like extension superiorly that may shed fluid in the newborn period. It is always present at birth, yet it might be neglected, missed, or misdiagnosed. The clinical and pathophysiological aspects, imaging findings, and surgical treatment of this unusual and seldom documented condition in a 9‐year‐old Nigerian girl are described.

## 2 CASE REPORT

The patient presented with an anterior midline cervical lesion that was discovered at birth but misdiagnosed as an iatrogenic wound caused during the baby's surgical delivery. History revealed that due to poor progress of labor, maternal preeclampsia, and potential fetal distress, the patient was delivered through emergency cesarean section. Despite frequent dressings with topical antiseptics, the lesion has not healed in the previous 9 years before presentation, this prompted her appearance to the surgical outpatient department. She is the only child of her parents. There is no family history of a similar neck lesion in both her parents and her extended family.

**FIGURE 1:**
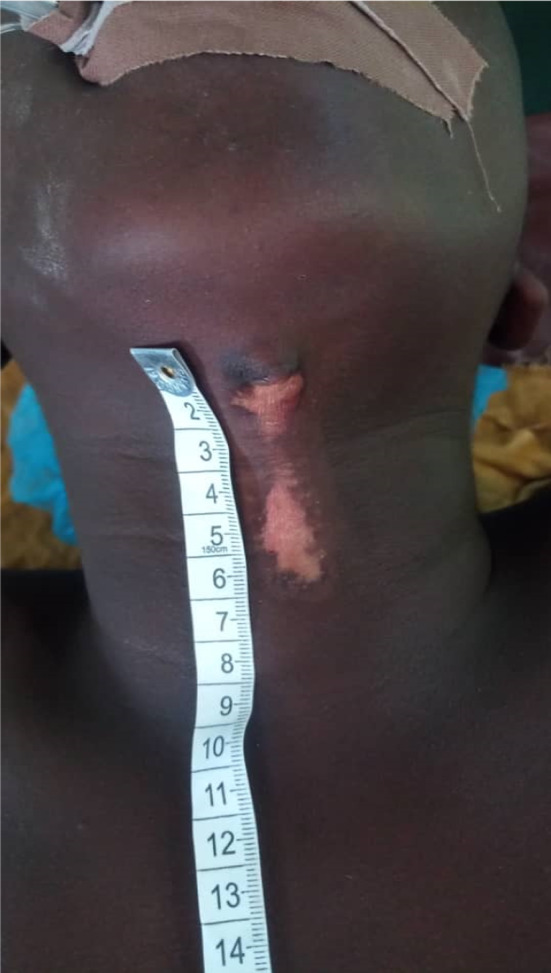
A linear lesion about 5 cm with a (3x3x1) cm papule at its superior end

On physical examination, an anterior neck lesion about 5 cm long, with a linear configuration, was observed. It runs from the submental region to the lower margin of the thyroid cartilage, with a (3 × 3 × 1) cm papule at the superior end (Figure 1). There was no movement of the nodule with tongue protrusion or deglutition.

When the neck was extended, a skin web was discovered between the cleft and the mandible. The physical examination showed no other congenital anomalies.

**FIGURE 2:**
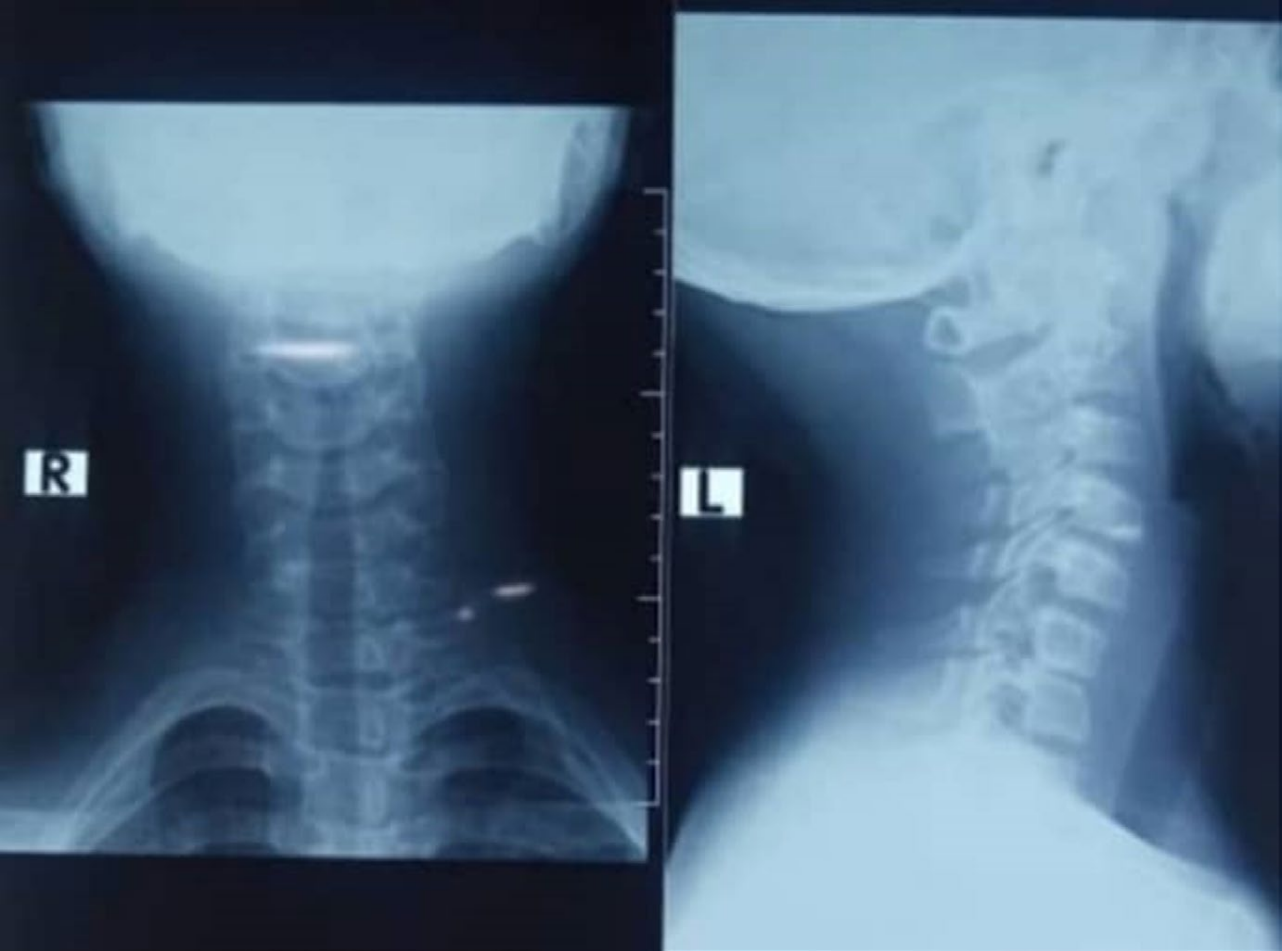
Plain X‐rays of the cervical spine (AP &) reveal the absence of intervertebral disc between the C6 and C7 vertebral bodies

Plain radiographs of the cervical spine in anteroposterior and lateral views were taken, and radiographs were displayed as shown in (Figure 2).

**FIGURE 3:**
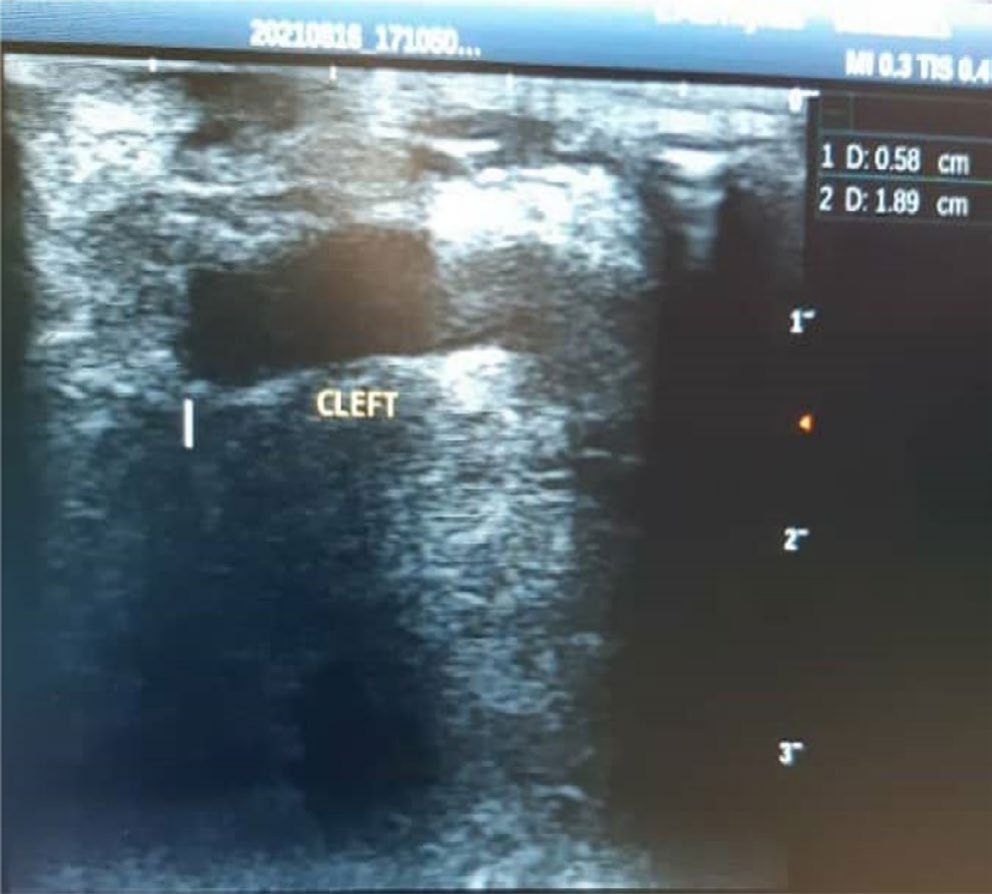
Neck USS revealing the presence of a cleft

Neck ultrasonography was also performed using the SonoScape equipment, with all pictures taken in B mode and a 7 MHz linear probe (Figure 3). This image reveals both thyroid lobes with normal parenchyma and the carotid sheaths in normal locations on both sides, ruling out any related thyroid abnormality. A cylindrical‐shaped cystic lesion with blind ends lying beneath the skin is shown as a focused longitudinal scan across the anterior neck lesion.

Parents were counseled on the need for her to have surgery because, at the time of presentation, and the patient exhibited some restriction in neck extension, which her parents observed was becoming more obvious as she grew older. The second reason of surgery recommended was that she was getting concerned about her appearance because she was becoming embarrassed by the frequent inquiry by many of her classmates as to why her neck appeared malformed or had a "wound" due to the dyschromic appearance in the midline of her neck.

The midline sinus/cleft was excised during surgery to achieve two goals: to eradicate the dyschromic midline cleft, thereby improving her appearance, and to arrest the gradual flexion contracture that the mother had noted.

**FIGURE 4:**
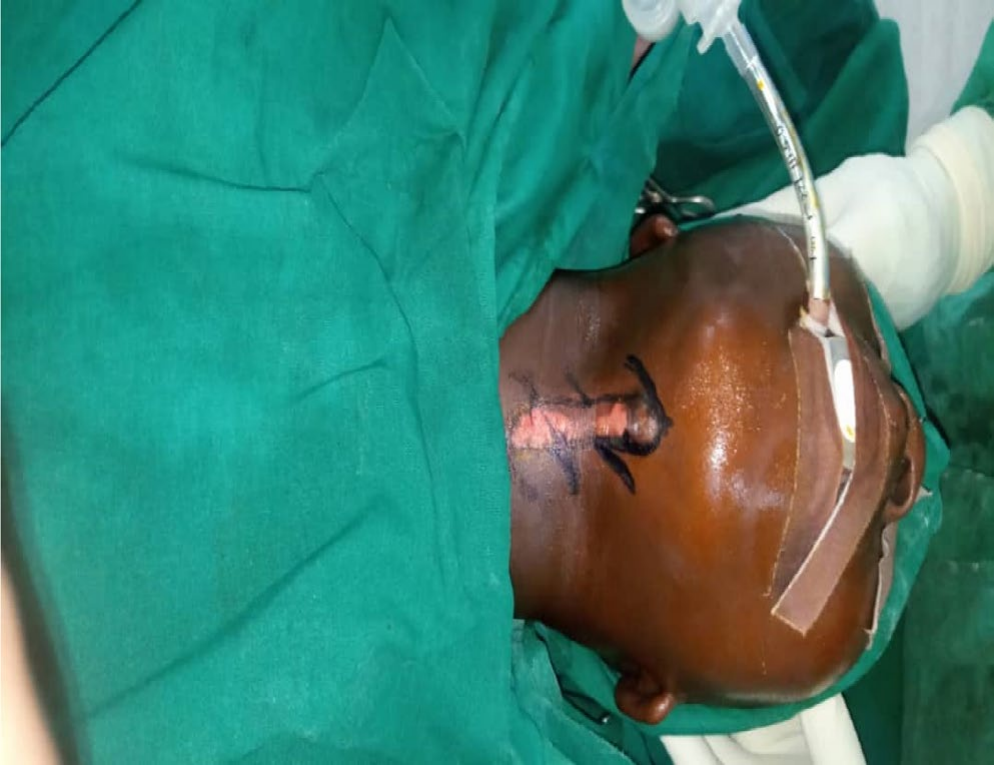
Multiple z‐plasty design was designed at the edge of the cleft

**FIGURE 5:**
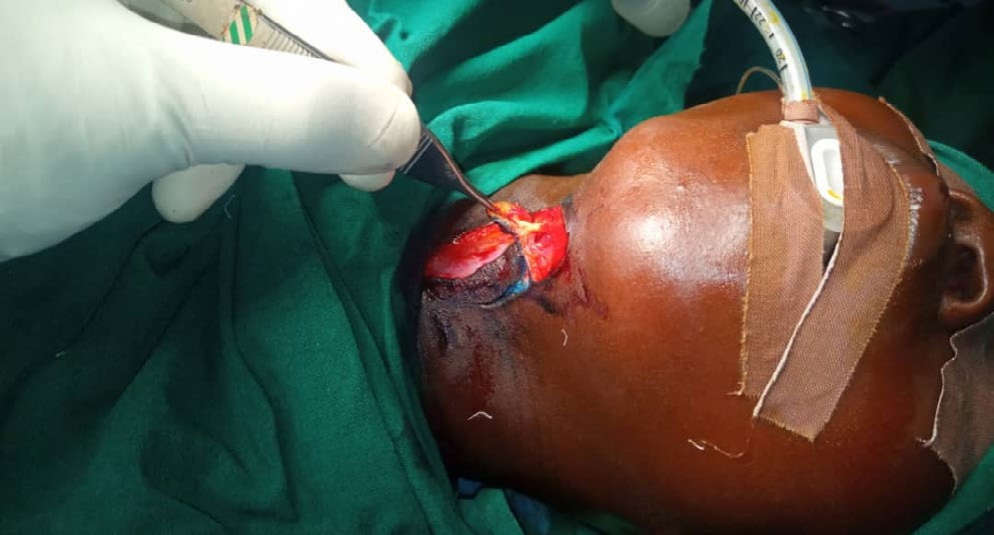
The incision revealed a (6 × 3) cm sinus tract extending inferiorly from the skin papule at the cephalic end

**FIGURE 6:**
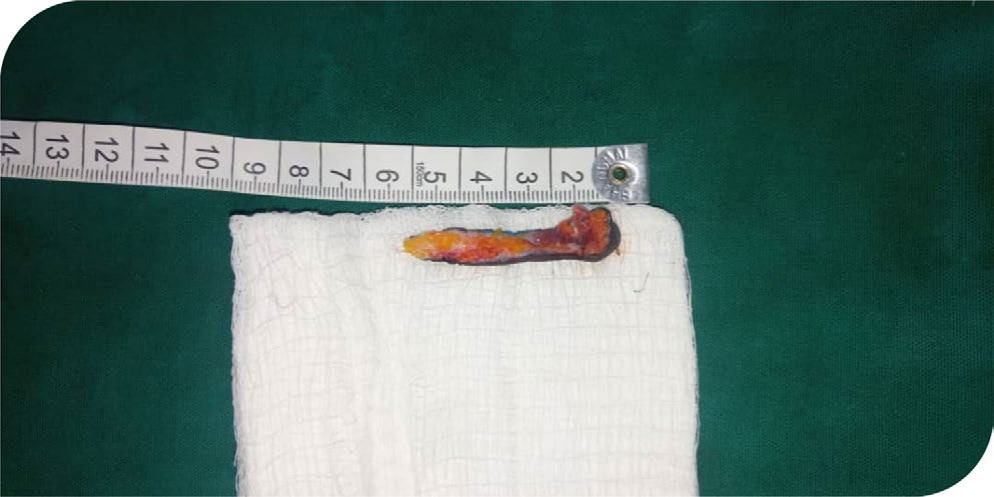
The excised sinus tract tissue

Under general anesthesia and endotracheal intubation, the patient was positioned in a supine posture with the neck slightly extended in the operating room with the aid of the head ring and shoulder support, in which neck‐band was more visible. As seen in Figure 4, multiple z‐plasty patterns were marked at the cleft's margin. The cleft was then injected with 1:200,000 diluted adrenaline. After about 7 min, incisions were made to excise the cleft, which included the superiorly positioned lump down to the dermis and some adjacent platysma muscle fibers. Though probing with a sinus probe revealed no evident sinuses, we included all visible clefts in our excision, as seen in Figure 5. The z‐plasty pattern that had previously been sketched at the cleft's margins was then incised, raised, and transposed. To avoid flap tip necrosis, the flap tips were carefully insetted with Gilles 3‐point corner stitches. Prolene 4‐0 and vicryl 4‐0 sutures were used for subcuticular dermal stitches. The wound closure was performed without tension. Dressings were applied after the procedure. Figure 6 depicts the tissue removed from the sinus tract.

**FIGURE 7:**
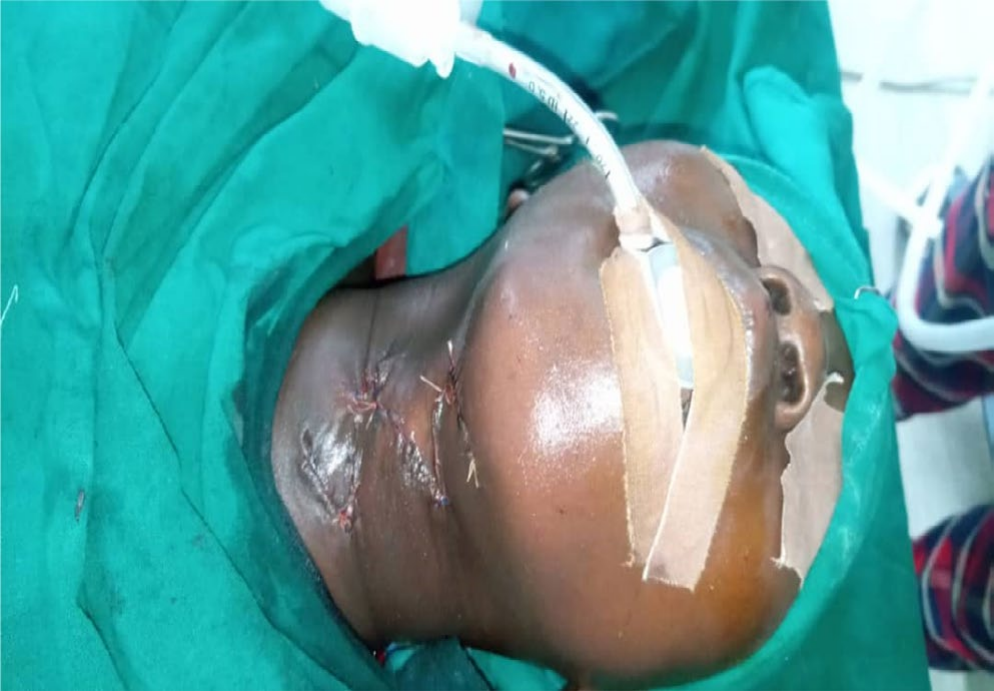
Closed wound in 2 layers using subcuticular absorbable stitches

**FIGURE 8:**
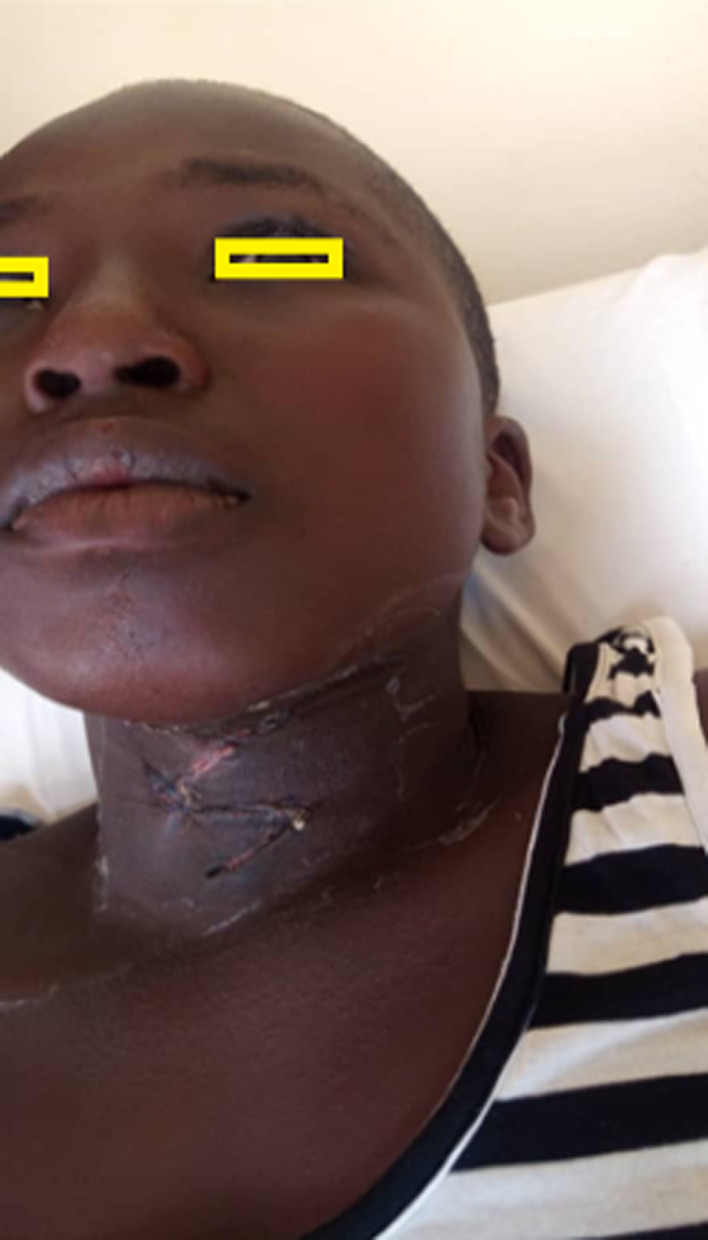
Wound healed well

Postoperatively, the patient made an uneventful recovery. The skin stitches were removed on the fifth postoperative day, and the wound edges were intact and healing nicely (Figure 7). She was discharged to the clinic for follow‐up. In the clinic, at follow‐up visit we noted the surgical site had healed well (Figure 8).

**FIGURE 9:**
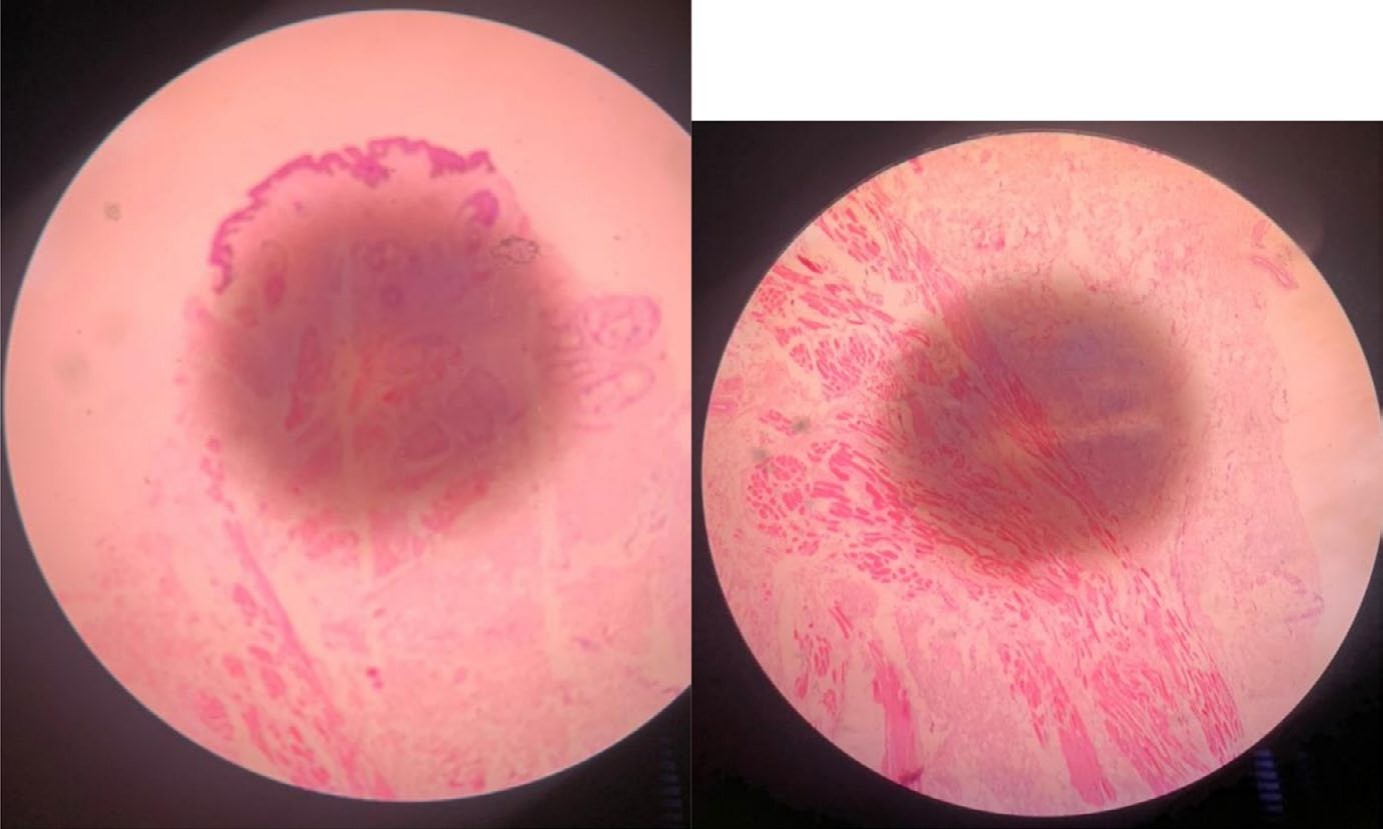
Histology of skin tag revealed include papillary hyperplasia with parakeratosis of the stratified squamous epithelium

The specimen's histology confirmed our clinical diagnosis. Histological findings included papillary hyperplasia with parakeratosis of the stratified squamous epithelium of the cephalic skin tag (Figure 9). The subepidermal layer was densely packed with pilosebaceous units, whereas the deeper layers were densely packed with striated muscle bundles. The mid‐portion and caudal end had no epidermis parakeratosis and no skin adnexal in the dermis. There was no duct or pseudostratified epithelium seen.

## 3 DISCUSSIONS

CMCC cleft has been variously referred to as CMCC cord, medial cleft, median neck fissure, midline cervical webbing, and pterygium coli medianum due to its morphological features.^1^ According to a literature search, it occurs in roughly 2% of all congenital neck malformations. By 2014, only about 205 cases had been recorded, and by 2018, only about 50 examples of this abnormality had been described in the English medical literature.^2^ Despite extensive study on congenital neck malformations, no specific cases of CMCC have been recorded from Nigeria. In Africa, the closest match is a 9‐year‐old girl reported in Egypt in 2018.^1^, ^2^, ^3^

In this case study, the patient had the classic CMCC findings: "a usually erythematous, vertical, and atrophic skin defect in the midline of the neck that lacks adnexal elements, a subcutaneous fibrous cord that is often longer than the overlying skin defect, a superior skin tag, and an inferior blind sinus."^4^, ^5^, ^6^

Many writers have postulated that the origin of CMCC is a failure of numerous embryological processes, which is detailed in some books.^6^ The inability of the branchial arches to fuse entirely at the midline is the most popular of these ideas, and it explains odd variants such as a cord without a cleft or the lack of hyoid bone and thyroid cartilage.^7^, ^8^, ^9^ Furthermore, an embryopathologic reason for the lack of hair follicles, sweat glands, or sebaceous glands in the cleft tissue is migration insufficiency. Others include a delay in mesoderm merging and a developmental field deficit affecting cervical midline parenchymatous structures, which might explain the lack of an intervertebral disc between the C6 and C7 vertebral bodies in the situation at hand.^6^

Wynn Williams proposed in 1952 that this abnormality was caused by a breakdown of circulation during development.^5^ Several writers have demonstrated that midline cervical clefts are linked with thyroglossal cysts, branchiogenic cysts, or other cystic diseases that cannot be explained by current etiologic ideas.^7^ CMCC is typically linked with a range of branchial arches‐related midline malformations, such as a median cleft of the lower lip and mandible, hypoplasia, or the lack of other midline neck structures.^8^ Furthermore, genetic variables such as SIX5 mutations and pregnancy‐associated plasma protein have been implicated in its pathogenesis.^5^ In three patients with isolated CMCC, a gene was discovered. "Both abnormalities were passed down from unaffected parents. These findings most likely indicate that the discovered mutations are not disease causing; however, they may be significant factors if CMCC is inherited in a polygenic manner. "^10^, ^11^, ^12^

A detailed physical examination of the patient is typically used to make the diagnosis of CMCC. The most common presenting ailment is a bump on the neck. Other differential considerations must, therefore, be kept in mind, and a neck ultrasound scan can be extremely useful in this regard. For a more accurate assessment of the soft tissue connections, computed tomography or magnetic resonance imaging may be used.

Delays in CMCC therapy impact the growth of the bottom region of the face, notably mandibular development and neck extension.^3^ To enhance the aesthetic look of the neck, CMCC is treated by total excision of the subcutaneous fibrotic band and underlying fibrous scar, followed by lengthening obtained by reconstructive multiple z‐plasty from the neighboring normal skin.^10^ Z‐plasty provides surgeons with the advantage of avoiding a straight‐line scar, which might result in retractile fibrous tissue and a bad esthetic outcome.^11^ Recurrence rates are significant, and cases have been observed up to 9 years following the first procedure.

Given the rarity of this illness, particularly in Africa, we believe it is important to record this instance.

Our findings are consistent with previous research. Our patient's postsurgery recuperation is going well.

## 4 CONCLUSIONS

Congenital midline cervical cleft is a rare congenital abnormality that healthcare providers should be aware of. This would allow for the early referral of patients to plastic and reconstructive surgeons for the appropriate and timely intervention required to enhance their results.

## CONFLICTS OF INTEREST

There are no conflicts of interest whatsoever.

## AUTHOR CONTRIBUTIONS

AIS, SKA, and BFO all contributed to the data gathering, analysis, and writing of the paper. TGO and AIO both contributed to the manuscript's composition and critical editing. AIS, SKA OAS, and KRA all participated to the care of this patient, data collecting, analysis, and writing and critical editing of the text. All authors read and agreed with the content of the manuscript.

## 5 ETHCAL APPROVAL

Our institution's ethics committee granted ethical approval. During this case report, the authors followed appropriate EQUATOR network (http://www.equator‐network.org/) standards, most notably the CARE guideline.

## 6 CONSENT

Written informed consent was obtained from the patient's parent to publish this report in accordance with the journal's patient consent policy.

## Data Availability

Data are available with the corresponding author and will be provided when and if necessary.
